# Annotations

**Published:** 1900-07-21

**Authors:** 


### Annotations.

### The Butchers and Tuberculous Meat.

We have heard so much lately about the prevention of tuberculosis, and so much has been done to disseminate knowledge far and wide as to the various modes in which the disease is spread, as to the steps to be taken for its prevention, and even as to the general principles underlying its treatment, that it must, we should imagine, come rather as a shock to those hopeful people who, because they live in a civilised country, imagine that "the authorities" will take care of them, to find that the very authorities in whom we all have to trust, and the very laws which we all take our share in making, still give their sanction to the public sale of the meat of tuberculous animals as food for man. How far the eating of tubercle-tainted food is responsible for the tuberculosis which prevails so widely we will not here discuss, but we will at least say that all those measures of which we have heard so much, which aim at preventing the disease by putting a stop to the dissemination of its infection are lamentably defective—nay, are grotesque in their incompleteness—so long as the public markets are open for the public sale, for the food of man, of the carcases of beasts which are known to be the subjects of tuberculosis. A case[1] which has recently made considerable stir, both in the meat trade and among sanitary authorities, serves well to illustrate the amount of tuberculosis which is sometimes passed by those to whom the duty of inspection is committed, as not interfering with the fitness of a carcase for sale as human food. The particular animal in question previous to slaughtering appeared healthy to the inspector, to the manager of the slaughterhouse, and to his assistants. After slaughter, however, it was found that both lungs contained numerous large and small tuberculous nodules and several abscesses filled with creamy pus, the lungs being "very badly diseased." The thoracic lymphatic glands contained numerous tuberculous nodules. The liver contained numerous tuberculous nodules, both large and small. The pharyngeal lymphatic glands were also affected with pronounced tuberculosis. The spleen also presented abnormal patches on the surface, but it was not ascertained whether or no these were the result of tuberculosis. The carcase appeared well fed and healthy to the naked eye. The question, then, was whether this was to be considered a case of generalised or of localised tuberculosis. Dr. Sykes, the medical officer of health of the district (St. Pancras) in which the slaughter-house was situated, came to the conclusion that the animal was suffering from general tuberculosis, and ordered the carcase to be detained. The owners were communicated with, and the carcase and offal were inspected by several veterinary authorities; and Dr. Sykes consulted Dr. Bond, medical officer of health for Holborn, in whose district the meat market at Smithfield is partly situated, as to the custom at that market. Dr. Bond agreed that the organs were affected as described, and remarked that the lungs were "very bad," but he could not detect tubercle with the naked eye in the lymphatic glands of the carcase, and he stated that under that condition the carcase would not be seized in the London Meat Market, but the internal organs would be destroyed. "This evidence as to local trade practice," says Dr. Sykes, "would in all probability have led a magistrate to decide against condemnation, and consequently very costly legal proceedings might have ensued. . . . I therefore gave instruction that the carcase should be released from the mutually agreed detention." We do not quote this case with any desire to criticise the action of Dr. Sykes, because we well know how vexed a question is involved, and how ready the butchers are to fight it out at law. All we wish to do is to show that, notwithstanding the recommendations of the Royal Commission on Tuberculosis, and all that is being done in many directions to put a stop to the dissemination of the infection of this disease, this is the sort of meat which is now "passed" on the plea that the disease is "localised." We quite agree with all that is being done by the National Association for the Prevention of Consumption. Each of its recommendations is right in its proper place. But is there not something farcical in the general "booming" of spittoons and of the various preventive measures which are now being preached so widely, the isolation of consumptive invalids, the boiling of milk, the abolition of dusters, and all the other things we are to do lest perchance a little microbe should enter our bodies, when all the time the sanitary authority appointed for the management of everything that concerns the health of one of the greatest of our London parishes is, through its officers, shaking in its shoes at the mere thought of what the butchers might do if they were crossed, and is allowing meat such as we have described to be supplied as food to the very people for whose protection in such matters the authority is by law established?

[1] The Meat Trades Jour., April 26, May 3 and 10; and Public Health, July, 1900.

### The Army Medical Department.

Now that the Government has undertaken that a thorough investigation shall be made into all the circumstances attending the treatment of the sick in South Africa one's feeling is strongly in favour of letting the subject alone until we at least obtain an authoritative statement of the facts. Still, when men in an official position speak it is impossible to avoid some sort of comment on their utterances, and in view of the extremely cheerful and optimistic speech made by Surgeon-General Jameson at the dinner of the Epidemiological Society last Friday, we must point out how characteristic of what one may call the departmental way of looking at things was his way of regard-

**ANNOTATIONS**—*continued.*

ing the subject. He is concerned for the great department of which he is the head; he believes that the Commission will bring out the truth; and he "will bet his last dollar that the Army Medical Department will come out with flying colours." That is the worst of officials; each sticks up for his own office. They forget that the British public does not care a brass farthing about the Army Medical Department except as an integral part of that fighting machine which we call the army, and that however perfect its arrangements within itself may be, it is a failure if its organisation, and its relation to the rest of the army, fail to ensure to the troops a reasonable freedom from disease. The question is not merely one of provision for the sick. Far more is it one of the sanitary arrangements for the campaign, and of the extent to which the organisation of our army permits and demands the due consideration of health questions in those inner councils which decide upon great military movements. It is all very well to lay the blame, as Surgeon-General Jameson did, on the infected water at Paardeburg, and thus to suggest that the outbreak of enteric fever which took place was inevitable, and such as could not have been foreseen or prevented; but a consideration of the weekly admissions into the hospitals at Bloemfontein is enough to show that it was not at Paardeburg that the mass of the fever was caught. It was brought up from there no doubt, the infection being then scattered about the camps around Bloemfontein, but it is to the fouling of these camps that we must look for the cause of the great outbreak of fever which took place later on. What we have to face is not merely that there were local defects in the distribution of the medical supplies, such as doctors, nurses, beds, &c., after the catastrophe arose, but that notwithstanding all our boasted advances in sanitation, the organisation of our army as a whole—including, of course, that of the medical department, and especially its relation with the fighting arm—has turned out to be such that it has proved impossible for our men to camp for a few weeks anywhere in South Africa without setting up a focus of typhoid fever. Too much has been said about marches. It is as a camp disease that typhoid has hit us so hard, and this is chiefly a matter of camp discipline.

**The British Medical Association.**

The growth of the British Medical Association, the important position which, by aid of its journal, it has taken in giving voice to the opinion of the profession, the widespread influence of its branches, scattered as they are throughout the British Empire, and, finally, the fact that with the accumulation of wealth the exploitation of its money bags has offered dangerous attractions, have combined to make it desirable that the rules by which it is governed should be revised and brought into accord with its present surroundings. An extraordinary meeting of the Association was held last Wednesday at Exeter Hall to consider a new set of regulations submitted by the Council with the object of amending and codifying the existing rules. It need hardly be said that the occasion had been taken advantage of to introduce certain fundamental modifications in the articles of association especially as to the powers of the general meetings. The best friends of the Association have long seen that if the powers of the general meetings were in reality what they were claimed by some to be, and if the general policy of the Association was to be dictated by the snatch votes recorded at those very unrepresentative meetings, the whole future of the Association would be in constant danger of falling into the hands of any noisy clique that could manage to get together a score or two of followers. It was therefore considered very desirable that some limit should be put to the vagaries of the general meetings, and that the management of the affairs and the policy of the Association should be given more completely than before into the hands of the Council. The Council is the direct representative of the branches, and it is in the life of its branches that the great central tree of the Association lives; and although much has been said about the Council filching the powers which have in theory belonged to the general meetings, everyone knows that the Council is a safe and steady-going body, which is far more to be trusted than any orator with his tag of followers at any general meeting. If people wish to influence the policy of the Association, it seems reasonable to ask them to share the heat and burden of the day in the conduct of its branches. The Exeter Hall meeting, however, would have none of this, and amid considerable confusion threw out the proposed reform.

**Experiments on Living Animals.**

As is well known, a large proportion of the cases reported as experiments on living animals—experiments for the performance of which licences and certificates are required, and for the supervision of which a Government official has been appointed in accordance with the terms of an Act of Parliament—are merely injections and inoculations. Now, during recent years, while the mere vaccination of rabbits and guinea-pigs has been so carefully hedged round with legal machinery, the much more serious and severe operation of injecting animals with tuberculin and with mallein has come into somewhat extensive use, the object of the operation being to ascertain whether or not these animals are the subjects of tuberculosis or of glanders. This operation is clearly an experiment upon a living animal. We believe that it has been decided that the inoculation of a horse with the poison of diphtheria, which would certainly be an experiment, and would require to be done under the ægis of the Vivisection Act, if it were undertaken with the object of increasing our knowledge of disease, can be done, and is constantly being done, without either supervision or restriction when it is performed as part of the trade process of manufacturing anti-toxic serum; for however harshly the law deals with science it deals almost as tenderly with trade as it does with sport. We can, however, hardly regard the experiment of injecting mallein to see whether a horse has got glanders, or that of injecting tuberculin with the object of ascertaining whether or not a beast is tuberculous, and thus of deciding what price should be paid for it, as part of a trade or manufacturing process. In both cases the proceeding is a pure and simple experiment on a living animal, and it would be interesting to know how the exemption of such experiments from the restrictions imposed by the Vivisection Act can be justified

